# Case report: Persistent hypogammaglobulinemia in thymoma-associated myasthenia gravis: the impact of rituximab or Good's syndrome?

**DOI:** 10.3389/fneur.2023.1152992

**Published:** 2023-05-05

**Authors:** Jingru Ren, Jianchun Wang, Ran Liu, Jing Guo, Yan Yao, Jingjing Luo, Hongjun Hao, Feng Gao

**Affiliations:** Department of Neurology, Peking University First Hospital, Beijing, China

**Keywords:** myasthenia gravis (MG), rituximab (RTX), hypogammaglobulinemia, infections, Good's syndrome (GS)

## Abstract

**Introduction:**

Rituximab (RTX) showed good efficacy and safety for patients with myasthenia gravis. However, the percentage of peripheral CD20+ B cell may be absent for years after low dose of RTX treatment. Persistent hypogammaglobulinemia and opportunistic infection may occur in patients under treatment of RTX with thymoma relapse.

**Case representation:**

We report a case of refractory myasthenia gravis. After two doses of 100 mg rituximab, the patient developed transient neutropenia. The peripheral blood CD20+ B cell percentage was 0 more than 3 years. Eighteen months later, the patient's symptoms relapsed with thymoma recurred. She had persistent hypogammaglobulinemia and multiple opportunistic infections.

**Conclusion:**

In MG patient under B cell depletion therapy had thymoma relapse, Good's syndrome may induce prolonged B cell depletion, hypogammaglobulinemia and opportunistic infections.

## Introduction

Many studies have indicated that rituximab (RTX) is effective for myasthenia gravis (MG) (1). Although most studies have suggested that rituximab is safe, serious adverse events have been reported, including severe skin reactions, blood system diseases, hepatitis B reactivation, progressive multifocal leukoencephalopathy and other severe opportunistic infections (2). Conditions may become more complicated in patients with MG and thymoma.

Herein, we report a patient who received rituximab and radiotherapy for MG with thymoma. She suffered from transient severe agranulocytosis, persistent hypogammaglobulinemia, opportunistic infection and haemolytic anemia.

## Case description

The patient is a 34-year-old female who was diagnosed with generalized myasthenia gravis 12 years prior. She underwent thymectomy and radiotherapy. Previously, she had three episodes of myasthenia gravis crises induced by infection. In addition to prednisone, she received azathioprine and cyclosporine, which failed to control her symptoms. Her dose of tacrolimus was halved due to the high blood concentration.

Physical examination showed bilateral mild ptosis, and the distal muscle strength of the lower extremities was Grade IV. She was positive for anti-acetylcholine receptor (anti-AChR) antibody. The myasthenia gravis Foundation of America (MGFA) classification was IIb, and the Activities of Daily Living (ADL) and Quantitative Myasthenia Gravis Score for disease severity (QMG) score was 11 and 15, respectively. The baseline total T lymphocytes, total B lymphocytes, CD20+ B cells and CD22+ B cells were 79% (50–82%), 9.6% (5–21%), 9.5 and 9.6%, respectively. The patient was treated with rituximab (100 mg) intravenously in February 2019. After discharge, she continued to take prednisone (20 mg) daily. After 3 months, the percentage of CD20+ B cells in the peripheral blood was 0.1%. She was given another intravenous infusion of rituximab (100 mg) in May 2019, and the number of CD20+ B cells decreased to 0 on the second day. Her symptoms were quite stable, and she was prescribed 60 mg of pyridostigmine bromide and 20 mg of prednisone orally for maintenance therapy.

One month later, the patient presented with intermittent low fever, night sweats and herpes around the mouth, accompanied by ptosis of the right eyelid, dysarthria and chewing difficulty. The peripheral blood leukocyte count was significantly decreased to 1.65 × 10^9^, and the neutrophil count was 0. Peripheral platelet count and red blood cell count were normal. The total peripheral blood B lymphocytes and CD20+ B cells were still 0. The immunoglobulin G (IgG) level was 8.51 (7.23–16.85) g/L. The immunoglobulin A (IgA) level was 0.78 (0.69–3.82) g/L. The immunoglobulin M (IgM) level was 0.83 (0.62–2.77) g/L. The patient's symptoms improved after empiric anti-infection treatment with cefatriaxone, acyclovir and colony stimulating factor. The peripheral counts of leukocytes and neutrophils returned to normal 3 months later. The dose of prednisone was tapered to 17.5 mg.

In August 2020, the patient's symptoms were aggravated. Her chest computed tomography (CT) showed that the thymoma recurred. She received intravenous infusion 20g of immunoglobulin (IVIG) for 5 days and 25 sessions of thymic intensity modulated radiotherapy, with total dose of 5000 gy in May 2021. Her immunotherapy regimen was adjusted to 20 mg and 12.5 mg of prednisone every other day, and her symptoms stabilized.

In August 2021, the patient had fever again. Her chest CT showed that both lungs had new multiple patchy ground glass foci. The percentage of total T lymphocytes was 96.5%, the percentage of CD4+ and CD8+ T cells was 23.7 and 72.5% respectively, and the percentage of B cells was still 0% in peripheral blood. The level of IgG was 29.3 g/L, the level of IgA was 0.49 g/L and the level of IgM was 0.58 g/L. Bronchoscopic alveolar lavage indicates pneumocystis pneumonia. IVIG (20 g) was administered intravenously for 5 days. Sulfamethoxazole tablets were given as treatment. Her prednisone dosage was reduced to 20 mg and 5 mg orally every other day. Two months later, the level of IgG was 7.86 g/L, the level of IgM returned to 0.65 g/L, and the level of IgA was still as low as 0.47 g/L.

In October 2021, the patient experienced decreased vision in her left eye, and she was diagnosed with cytomegalovirus infectious retinitis. Additionally, the IgG, IgM and IgA levels were all below normal limits (6.86 g/L, 0.43 g/l and 0.51 g/L). The patient received ganciclovir intravitreal injection, tobramycin and prednisone acetate eye drops, and the left eye vision of the patient was maintained at 0.15. Steroids were discontinued, and 20 g IVIG was administered every month. After 5 administrations, the interval was extended to 2 months, and the patient's immunoglobulin level was monitored monthly. The IgG level returned to normal, but IgM and IgA levels fluctuated between 0.45–0.5 g/L (0.63–2.77 g/L) and 0.27–0.4 g/L (0.69–3.82 g/L), respectively, for a long time.

**Figure 1:**
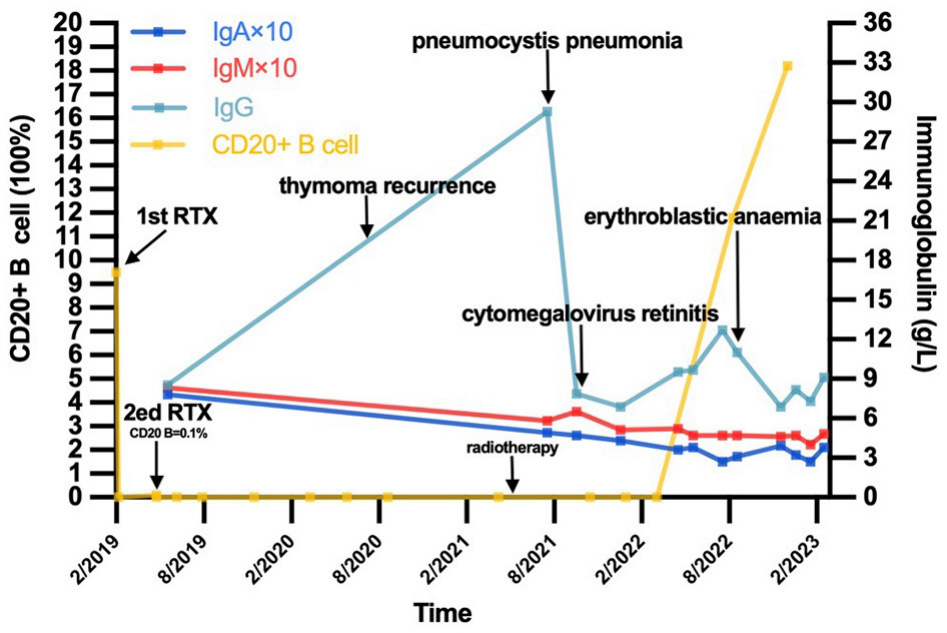
The timeline of this patient's course and the trend of CD20+ B cell as well as immunoglobulin level. IgA, immunoglobulin A; IgM, immunoglobulin M; IgG, immunoglobulin M.

In May 2022, the percentage of peripheral CD20+ B cells was 11.65% (5.0–21%). The patient's myasthenia symptoms were relatively stable, leaving only extraocular muscle paralysis and mild limb weakness. In August 2022, however, the patient felt fatigue and dizziness. Routine blood tests revealed anemia. HGB levels decreased to 50 mg/dl at minimum. Coomb's test was positive, and the patient was diagnosed with erythroblastic anemia. IVIG was given with 40 mg prednisone. HGB levels returned to 94 mg/dl. At present, the patient's symptoms are quite stable except for low immunoglobin levels. Her IgG level was 6.87 g/L, IgA and IgM was 0.39 g/L and 0.46 g/L, respectively. Her corticosteroids were tapered slowly, and IVIG was administered every 2–3 months. We summarized the patients clinical events and laboratory results in Figure 1.

## Discussion

In the current case, transient neutropenia was induced by RTX and persistent hypogammaglobulinemia preceded by thymoma relapse, resulting in multiple opportunistic infections.

The incidence of neutropenia has been reported to be approximately 6.5% after RTX treatment in patients with rheumatoid arthritis. Most patients have a good prognosis without severe infections (3). Due to the low dosage of RTX and the patient was asymptomatic, the level of immunoglobin was not evaluated before and after treatment of RTX. Three months after thymoma relapse and radiotherapy, hypogammaglobulinemia was found, while peripheral B cell was still 0. These leads to multiple opportunistic infections. Both RTX and thymoma can induce hypogammaglobulinemia.

The incidence of hypogammaglobulinemia following RTX treatment ranges from 13 to 56% in patients with non-Hodgkin lymphoma and autoimmune diseases, which is more common and severe than neutropenia (4, 5). Most studies believe that persistent IgG deficiency is linked to an increased risk of serious infections after RTX and that decreased IgM and IgA are less clinically significant (5–8). At present, the etiology of hypogammaglobulinemia after RTX has not been fully clarified, and many studies believe that it is the result of the interaction of multiple factors (9). RTX mainly acts on the surface antigen of CD20, which is mainly expressed in peripheral blood circulation B cells but not bone marrow stem cells and CD20-negative plasma cells (10). Therefore, RTX will not completely inhibit humoral immunity. However, in patients with haematologic diseases, combined chemotherapy may lead to persistent hypogammaglobulinemia related to the stagnation of B-cell reconstruction (11). It is unclear whether the differences in dosage and interval of RTX will have a variable impact on the degree and duration of B-cell depletion (12, 13).

Theoretically, with the regeneration of peripheral blood CD20+B cells, the immunoglobulin level should return to the normal level. After two doses of rituximab 100 mg, the level of B lymphocytes in this patient remained at 0 for 3 years. As the level of IgG returned to normal, the levels of IgA and IgM in peripheral blood continued to be lower than the normal limit, even after CD20+ B cells returned to the normal level. There might be other factors influencing immunological function.

Immunological disturbances such as Good's Syndrome may occur in patients with thymoma, including a reduction in circulating B lymphocytes, hypogammaglobulinemia and decreased T lymphocytes (14), presenting with a variable phenotype of immunodeficiency. The current patient had persistent hypogammaglobulinemia, B-cell deficiency and thymoma, Good's syndrome should be considered. Decreased T lymphocytes, inversion of the CD4+/CD8+ ratio also occurred in this patient (11). Comparing with the treatment of RTX, Good's syndrome is more accountable for the subsequent immunodeficiency.

At present, there are no proven diagnoses or treatments. Immunoglobulin replacement reduces the risk of infection with primary and secondary immunodeficiency associated with hypogammaglobulinemia. Some studies believe that the clinical manifestations of severe infection are more reliable as indicators of alternative treatment than laboratory indicators (15). The B-cell phenotype of patients receiving immunoglobulin replacement therapy after rituximab is mainly naive B cells, and the switching memory B cells are reduced (16).

## Conclusion

In MG patient under B cell depletion therapy had thymoma relapse, Good's syndrome may induce prolonged B cell depletion, persistent hypogammaglobulinemia and opportunistic infections.

## Data availability statement

The original contributions presented in the study are included in the article/supplementary material, further inquiries can be directed to the corresponding author.

## Ethics statement

Written informed consent was obtained from the individual(s) for the publication of any potentially identifiable images or data included in this article.

## Author contributions

JR and RL analyzed data and wrote the main manuscript text. JW and JL followed up patients and collected data. JG and YY examined immunoglobulin and lymphocyte level in serum. HH helped interpret relevant indicators. FG provided ideas and guidance for this article. All the authors read and approve the final revision of the manuscript.
